# Resveratrol: A Fair Race Towards Replacing Sulfites in Wines

**DOI:** 10.3390/molecules25102378

**Published:** 2020-05-20

**Authors:** Emmanouil Kontaxakis, Emmanouil Trantas, Filippos Ververidis

**Affiliations:** Plant Biochemistry and Biotechnology Group, Laboratory of Biological and Biotechnological Applications, Department of Agriculture, School of Agricultural Sciences, Hellenic Mediterranean University, GR 710 04 Heraklion, Greece; kontaxakis@hmu.gr (E.K.); mtrantas@hmu.gr (E.T.)

**Keywords:** resveratrol, sulfites, functional wine, sulfur dioxide, antioxidant, health effects, metabolic engineering, wine making

## Abstract

In recent years, significant efforts to produce healthier wines has led to the replacement or reduction of the addition of sulfites, using alternative substances or techniques. Resveratrol and related biophenols seem to be of great interest, since beyond their protective nature and contrary to sulfites they can positively affect consumer health. These bioactive phytochemicals are naturally produced in grapes as evolutionary acquired mechanisms against pathogens and UV irradiation. However, despite the efforts made so far attempting to develop economic and industrially adopted isolation techniques, available quantities of these biophenols for commercial use are still quite limited. Therefore, such molecules are still not able to meet the needs of industrial use due to their prohibitive marketable cost. In this review we summarize the efforts that have been made to biosynthesize these molecules through alternative, innovative ways. Increasing interest in modern biotechnological approaches has shed light on the exploitation of metabolically engineered microbial factories, instead of plants, to produce molecules of industrial interest. Such approaches, also reviewed here, are expected to lower the cost and appear promising to produce enough surplus to attract further oenological experimentation upon yielding functional wines. This development is expected to attract further industrial attention, continuing the race to partially or totally replace the external addition of sulfites. We also review important physicochemical properties of resveratrol in relation to enriching wines.

## 1. Introduction

Wine, although directly related to wellbeing and social life in general, mainly differs from other pleasurable alcoholic drinks in relation to its composition of natural substances. Because of this, there is growing evidence that when it is consumed in doses legally regarded as safe, it can be advantageous to health [1]. Moreover, contrary to the current economic difficulties worldwide, the wine market economy is expanding quite rapidly [2].

The *trans* isomer of resveratrol is the only functional conformation that has been linked to beneficial properties [3]. As a natural plant substance, resveratrol has gained an extremely positive reputation in the past decade(s) [2,4], particularly for its health-related properties, such as in the prevention of atherogenesis [5], cardiovascular diseases [6], oxidative stress in neurodegenerative diseases [7], and cancer chemoprevention cases [8]. Being colorless, contrary to other phytochemicals derived from the same phenylpropanoid metabolic pathway (e.g., anthocyanins or flavonoids) of secondary metabolism [9,10], resveratrol has been biosynthesized through biotechnological means [10,11]. Its connection to health and longevity through moderate wine consumption [7] began with the French paradox issue [6,12] that has been greatly attributed to cardiovascular disease protection, as red wine particularly has a rich content of bioactive phenolics [13]. Moreover, resveratrol and related biophenols are generally well tolerated in humans and animals [14] when administered as drugs in high doses in clinical trials, with some side effects recorded when tested to combat diseases previously mentioned [15]. These important health claims partly explain the fact that resveratrol is probably the most investigated plant secondary metabolite [16].

Wine-makers use sulfites in various steps of winemaking, from fermentation to bottling [17]. However, sulfites have been connected to human health problems [18], and despite the various attempts to replace or minimize their levels in the winemaking process, no significant and widely used commercial process with marketable proof has appeared so far.

In this review, we summarize research data and present considerations concerning the production of wines with reduced or no added sulfites by replacing them with resveratrol as a very successful candidate [19]. We also consider the production of such natural wines that can promote the need of consumers to be able to safely consume wine as a health-protecting functional product, without showing any allergenic or other pathologic side effects.

## 2. Wine Sulfites and Human Health

It has long been known that *Saccharomyces cerevisiae* can use sulfate, sulfite, or elemental sulfur as a source of sulfur to synthesize sulfur-containing amino acids (methionine and cysteine) [20]. Thus, wine yeasts are responsible for the liberation of sulfites, resulting in their bio-accumulation, depending on the environmental conditions and yeast fermentation activity [21,22]. These levels can range from 10 to 20 mg/L of “bound” sulfite in wine and in certain cases can reach 30 mg/L. Therefore, it is nearly impossible to produce wines without sulfites, even if no sulfite is added [23].

In the ancient Greek and Roman winemaking history, the external use of sulfur is documented as a common practice to preserve wine [17,21,22,24]. Moreover, sulfites are also added as antimicrobial agents [25]. However, the extensive use of sulfites resulted in the emergence of incidents of intolerance that included headaches [26], behavior disturbances, skin rashes, and other symptoms [23]. This resulted in the formation of specific legislation to control sulfite levels in final products [1]. This legislation intended to regulate and monitor the upper allowed limits of sulfites [27] and helped to standardize oenological methods in terms of lowering sulfite concentration in wines that would raise health-related objections as well as symptoms [23].

Improved winemaking protocols and shifting consumer habits towards health consciousness [28] has pushed winemaking to more sustainable production systems able to exert special characteristics [29]. Newly formed consumer habits include the selection of labels from innovatively-produced wines made through eco-friendly protocols [30]. In this way the wine market expands towards the production of wines with lower levels or even without additional sulfites, but retaining the organoleptic characteristics [29,31].

The World Health Organization has set the recommended daily allowance (RDA) of sulfur dioxide at 0.7 mg SO_2_/kg of body weight, estimating the acceptable intake of a 70 kg individual as 49 mg per day [20]. The upper limit allowed by the EU is 150 mg/L in dry red wines and 200 mg/L for dry white wines (Table 1). Consequently, even the consumption of half a bottle of wine (375 mL) can reach an amount of SO_2_ higher than the RDA. This might be one of the reasons why national and international health authorities demand additional decreases in the legal limits of SO_2_ [23]. The directive for the decrease in sulfite concentration is also dependent on the fact that even moderate wine consumption may cause severe headaches, depending on consumers’ sensitivity, which have been found to directly relate to external sulfite addition or even occasionally to the internally produced concentrations [26].

Consequently, due to the health-related side effects attributed to sulfur dioxide (SO_2_), even at regular levels appropriate labeling is required. Recent market research [30] shows that consumers have developed health-consciousness concerns that drive them to read the bottle label before selecting a wine product. Thus, legal measures are now considering a labeling act for each wine product, making it easier for the consumer to identify such risks before consumption. Such labeling offers protection to the high-risk group of people who suffer from chronic pulmonary diseases, such as asthma, that can easily evolve to bronchospasm [28,32].

According to some researchers, SO_2_ is the only additive that can provide a solution to wine preservation [28]. However, consumers have shown to be positive about adopting alternatives to sulfites, providing that they can also have a significant impact on their health and nutrition [33]. Recent research in the Italian wine market shows that consumers have developed an interest in choosing a wine with health-related claims [30]. The same reason also led consumers to choose organic wines with no added or lower sulfites, even at a higher price [31,34]. Organic wine production processes promote the limited use of chemicals, including sulfites, that are potentially harmful to human health. Such approaches involve the exploitation of selected indigenous winemaking yeasts in an effort to reduce the production of sulfites and derivative molecules, while targeting the balancing of the organoleptic quality of the wines that attain an important local flavor and character [35]. Recently, efforts have also been made to replace sulfites with alternative consumer-safe, as well as environmentally sustainable, bioactive compounds of plant origin, or by promoting winemaking with innovative techniques [36].

## 3. The Use of Sulfur Dioxide (SO_2_) in Winemaking

Sulfur dioxide or sulfurous anhydride (also known in winemaking by the term “sulfites”) is a colorless gas with important antiseptic and antioxidant properties. It is the most widely used preservative in the food industry [37], especially in winemaking where it has been used systematically since the 19th century [17]. Sulfur dioxide is of great importance in vinification as it protects against oxidation, inhibits the growth of “unnecessary” microorganisms, promotes the growth of selected yeasts [38,39], improves the release of phenolic compounds from grape skins and seeds during maceration, and stabilizes the color of the wine during aging [40].

Sulfur dioxide is available in several forms, as a gas, as an aqueous solution, or as salts (e.g., sodium metabisulfite or potassium metabisulfite) [41]. Winemakers may use SO_2_ at the stage of grape crushing, during the storage of wine in tanks or barrels, after the end of fermentation, or before bottling, in order to protect the wine from oxidation and microbial infections [17]. However, even without the addition of sulfur dioxide, an amount of SO_2_ is present in the wine after the end of fermentation as a result of amino acid metabolism in yeasts [42]. The amount of SO_2_ naturally present in wine depends on the strains of the yeasts and the conditions during fermentation [43]. A concentration between 0.6 and 0.8 mg/L of molecular SO_2_ is required for the microbial stability of wine, while 20–40 mg/L of free SO_2_ (as bisulfite, HSO_3_^−^) is required to delay or prevent wine oxidation [42].

Although various mechanisms have been proposed to explain the antimicrobial effect of SO_2_ [44], the most probable is the degradation of the disulfide bonds of the microbial proteins that leads to loss of function; bisulfite can bind to nucleic acids and lipids, thus causing damage to the microbial membranes [42,44]. As for its antioxidant action, SO_2_ is preferably oxidized instead of other compounds of must or wine, thus protecting its quality by retaining desirable organoleptic characteristics [41]. Moreover, SO_2_ also reacts with the byproducts of oxidation and suppresses the activity of non-enzymatic oxidative reactions as well as the activity of oxidases (such as the polyphenol oxidases), which are responsible for the oxidative browning of musts [17,42].

## 4. Disadvantages of the Use of Sulfur Dioxide in Winemaking

Despite the beneficial properties of SO_2_ in winemaking, the addition of SO_2_ presents several disadvantages, influencing consumers’ health and preferences. Excessive use may produce unpleasant flavors and aromas, or cause a cloudy appearance during storage [40]. The adverse effects of SO_2_ on consumer health makes labeling mandatory, although the maximum SO_2_ concentration allowed in wines is gradually being reduced [45]. The content depends on several factors, such as the type of wine and the production conditions such as noble rot wines and sweet wines from dried grapes [46]. Moreover, several pre-harvest practices and winemaking techniques that decrease the risk of wine being exposed to undesirable factors (e.g., bacteria, oxygen) can reduce the need for SO_2_ addition [47]. The maximum permitted amounts of SO_2_ in wine in the major wine-producing regions worldwide are presented in Table 1.

## 5. SO_2_ Replacement in Wines

In an effort to replace or reduce SO_2_ in wines, a wide range of natural or synthetic compounds as well as innovative winemaking techniques have been studied for their ability to protect the wine from oxidation and microbial contaminations, while preserving its organoleptic properties [40]. Several substances with antimicrobial activity, such as lysozyme, sorbic acid, dimethyl dicarbonate, are already permitted and used in winemaking, whereas other substances, although studied and found to have antimicrobial activity (such as bacteriocins, silver nanoparticles, hydroxytyrosol, fatty acids, yeast killer toxins, and antimicrobial peptides) are not yet permitted [40,48,49]. Similarly, in attempts to replace the antioxidant activity of SO_2_, various substances have been studied, such as ascorbic acid, glutathione, and a wide range of phenolic substances (e.g., tannins, resveratrol) [17].

On the other hand, several innovative physical methods/techniques have been tested, such as high hydrostatic pressure, ultrasound, ultraviolet irradiation, pulsed electric fields, and microwaves, aiming for the microbiological stabilization of wines through the reduction of the microbial load (yeasts, bacteria) of must or wine [17,49]. To adequately protect wine from oxidation or microbial spoilage, the combination of substances, specific winemaking methods, or the application of SO_2_ at reduced concentrations may be required [48].

In the following sections, studies showing the beneficial effects and possible drawbacks of using resveratrol to replace the externally added sulfites in wines either fully or partly are presented. We discuss how resveratrol may enhance wines, and what it can offer to the production of functional wines of high-quality.

## 6. Resveratrol Content in Wine

Resveratrol and other relative phenolic substances are naturally present in wines, as they get extracted from the crushed grape berries, skins, and seeds during must preparation and fermentation. However, the extraction process is time limited, and most of the phenolics are recovered in the byproducts of vinification and pomace of wineries [50]. The restricted amount of time that grape marcs remain with the must during fermentation in the process of making white and rosé wines is the main reason why those wines contain less phenolic substances, including resveratrol, as compared to red wines [51]. However, the resveratrol concentration in grapevine varies in concentration in different types of tissues and among white, red, and blue varieties of *Vitis vinifera* [52]. Moreover, the resveratrol content in wines depends on many factors, such as the grape variety, abiotic and biotic factors, cultivation, and oenological practices [53]. Thus, it is impossible to predict the resveratrol concentration, which can usually range from 1 to 4 mg/L, but may also be found in quantities greater than 10 mg/L [51]. In Table 2 we summarize the resveratrol content of white, rosé and red wines in the major winemaking countries worldwide. The data shown in Table 2 are presented separately for each extraction methodology, and grouped by country, as there is significant variation among the different isolation protocol surveys.

Several pre-harvest techniques have been studied or developed to increase the resveratrol content in grapes and therefore in produced wines. Stress factors applied during maturation of grapes, including infections, ultrasonication, visible or UV irradiation, as well as changes in the nutrition of plants, have been found to induce the biosynthesis of resveratrol in grapes [53,66]. Alternative extraction methods, modification of vinification parameters, thermovinification, extended maceration, must-freezing, and the addition of pectolytic enzymes have been applied for enhanced resveratrol and other phenolic substances content from grape skins and seeds [67,68]. Moreover, utilization of different yeast strains has also been found to significantly affect the resveratrol content in wines [69,70]. However, it must be noted that prolonged extraction may lead to an increase in substances such as tannins that may alter the organoleptic characteristics of wine (astringency).

The above-mentioned methods can be used to enhance resveratrol and relative biophenols in wines for its protection when SO_2_ is absent or in low concentrations. Nevertheless, the natural resveratrol content in grapes is too low to provide adequate protection for wines alone [48]. Thus, this information gives the possibility for wine to be fortified with higher levels of resveratrol obtained either from plant sources or biosynthesized from alternative means, as analyzed below.

Pastor et al. experimented with the addition of resveratrol at concentrations of 150 mg/L and 300 mg/L to study whether it could substitute SO_2_ in the vinification process with Cabernet Sauvignon [71]. The produced wines had similar physical, chemical, and organoleptic properties when compared to wines produced with the addition of potassium metabisulfite (7 g/100 Kg). However, the effect of resveratrol on the preservation of wine over time has not been studied. In a another study of Gaudette and Pickering [72], Riesling and Cabernet Sauvignon wines were enriched with 20 mg/L and 200 mg/L of resveratrol after fermentation. The concentration of resveratrol in the bottle remained stable for 58 weeks. The primary chemical indicators of wine quality were not affected by the addition of the resveratrol, while sensory changes were minimal. The antioxidant capacity of wines increased, and the color of Cabernet Sauvignon was also improved.

However, studies dealing with elevating the levels of resveratrol in wines focus mainly on the production of enriched wines as a functional beverage, due to its additional health benefits, rather than as a preservation treatment of the product itself.

## 7. Positive Effects of Resveratrol Intake on Consumer Health

The antimicrobial and antioxidant properties of resveratrol have attracted excessive interest from researchers that are experimenting to exploit its potential in human health. This partly relates to the so-called “French paradox”, which refers to the observation that despite the high levels of dietary saturated fat and cigarette smoking in France, the mortality rates from coronary heart disease are low. This has been attributed to the moderate consumption of red wine and its phenolic content, including resveratrol [13,73]. The intake of resveratrol through the consumption of food as well as through dietary supplements has been found to have a positive impact on the prevention and treatment of several diseases including cardiovascular diseases [74,75], several types of cancers (e.g., skin, breast, prostate, colorectal, liver, pancreatic, lung, esophageal, thyroid) [76], obesity and diabetes [77,78], and neurodegenerative impairments (Alzheimer′s, Huntington′s, and Parkinson′s diseases) [79,80].

The minimum daily intake to ensure the benefits of resveratrol is estimated to be 1 g per day and is relatively safe in doses up to 5 g [81]. At higher doses, some side effects may occur, although they are considered mild compared to the positive health benefits [82]. Nevertheless, the moderate daily dosage has been found to be better taken in a longer period of time than administering a single higher dose, as studies have shown that it is excreted from the human body within the first four hours after ingestion [81].

Among the foods with high resveratrol content, red wine is the primary source. Yet, the intake of the required daily amount (>1 g per day) is impossible through the consumption of wine or any other food [73]. Thus, the production of “functional” wines, with enhanced resveratrol content, has been studied as an alternative, more efficient source of resveratrol intake. Those wines could provide consumers with higher amounts of resveratrol while reducing alcohol consumption, which may have adverse effects on their health [83].

In a study conducted in Spain by Barreiro-Hurlé et al. [84], regarding the prospect of consumers choosing a resveratrol-enriched red wine over a conventional one, it was found that there is room in the market for functional wines for which consumers are willing to pay more. However, despite the widespread acceptance of the beneficial properties of resveratrol-enriched wines, the possibility of being labeled as “functional” is restricted by the legislation of most countries because of the alcohol content [85].

## 8. Antioxidant Activity of Resveratrol in Wines

Oxidative rancidity is a significant factor for the deterioration of wine quality, causing undesirable organoleptic characteristics and producing substances dangerous to human health, so the use of antioxidants is considered crucial to prevent these side effects [86]. Sulfur dioxide is the most widely used substance in winemaking due to its antioxidant properties. However, its undesirable properties and consumer concerns, as analyzed above, direct attention to the use of alternative natural products [45].

Resveratrol, as well as several other phenolic substances naturally found in wine (e.g., gallic acid, quercetin, rutin), has been linked with significant antioxidant and radical scavenging activity [87,88,89]. At appropriate concentrations, resveratrol or/and relative phenolic substances can protect wine from oxidation. Murcia and Martinez-Tome [90], in a comparative study of resveratrol and other common food additives, found it to be a potent antioxidant compound. Furthermore, Gülçin [91], in a similar study with resveratrol, found it to be an effective antioxidant in comparison to other antioxidant compounds such as butylated hydroxyanisole, butylated hydroxytoluene, α-tocopherol, and Trolox, by using various in vitro antioxidant assays.

## 9. Antimicrobial Activity of Resveratrol in Wine

Resveratrol has been found by many studies to have multiple health benefits, including antibacterial, antiviral, and antifungal activities [92,93]. However, these antimicrobial properties are essential not only for consumers but also for the protection of the wine. Several fungi and bacteria responsible for wine spoilage may be controlled when wines are supplemented with resveratrol or other relative phenolic substances, as a replacement or additionally to SO_2_.

Garcia-Ruiz et al. [94], in a comparative study of the inhibitory effects of wine polyphenols on the growth of lactic acid bacteria, found a significant antimicrobial activity of resveratrol against three lactic acid bacteria associated with the winemaking process: *Oenococcus oeni*, *Pediococcus pentosaceus*, and *Lactobacillus hilgardii*. In a similar study, Pastokorva et al. [95] found a significant antimicrobial activity of resveratrol against the yeasts *Dekkera bruxellensis*, *Hanseniaspora uvarum*, *S. cerevisiae*, *Zygosaccharomyces bailii, Zygosaccharomyces rouxii*, as well as against the acetic bacteria *Acetobacter aceti*, *Acetobacter oeni*, and *Acetobacter pasteurianus*.

Sabel et al. [96], in a recent study on antimicrobial properties against wine-related yeasts, lactic acid, and acetic acid bacteria, confirmed the antimicrobial activities of previous studies. However, besides undesirable microorganisms, resveratrol has also been linked to inhibitory effects on the yeasts *S. cerevisiae* and *S. bayanus*, which means that high doses of resveratrol, necessary for the protection of the wine, could affect the fermentation process. An inhibitory effect on *S. cerevisiae* was also found by Filip et al. [97]. Nevertheless, according to other studies, resveratrol was found to increase the life expectancy of *S. cerevisiae* in vinification through the activation of Sirtuins which are shown to regulate important biological processes like energy expenditure [98,99].

The minimum concentration of resveratrol for the inhibition of wine-related microorganisms is presented in Table 3.

In all cases, the doses found to be effective against the studied microorganisms were much higher than those found naturally in wines. The adequate protection of the wines, using resveratrol as the only antimicrobial agent, requires the addition of the substance. Thus, the solubility of the resveratrol could be a limiting factor, since it is characterized as low.

## 10. The Solubility of Resveratrol in Wine as a Limiting Factor for Its Industrial Application

Resveratrol is a lipophilic bioactive compound whose solubility in water is limited [100]. In wine, the solubility is increased due to its alcohol content, but it still remains limited [97]. In a naturally produced wine, where the resveratrol content usually ranges from 1 to 4 mg/L [51], the solubility is not a limiting factor. However, in the case of fortification of must or wines with resveratrol to increase their antioxidant and antimicrobial activity, solubility appears to play a significant role.

As shown in studies to determine the antimicrobial activity of resveratrol against undesirable fungi and bacteria that occur in wine, the applied concentrations range from 250 mg/L to more than 1000 mg/L (Table 3) [94,95,96]. Moreover, for the production of functional wines whose moderate consumption (1 glass of 150–180 mL) will provide the recommended daily intake (1 g of resveratrol) to ensure all the above-mentioned health claims [81], it is estimated that a concentration of more than 5000 mg/L is required. These quantities cannot be applied in practice, as the solubility of resveratrol in wine is limited to 30 mg/L in water [101]. Solubility is also dependent on temperature and pH, although the latter may negatively affect solubility only in alkaline solutions [102].

The most important factor that affects the solubility of resveratrol in wines seems to be the alcohol content. According to a study by Filip et al. [97], 40 mg/L of resveratrol was dissolved in 10% *v/v* ethanol (in water, at 20 °C). The solubility was significantly greater only at ethanol concentrations higher than 30% and at higher temperatures, conditions which are not easily met; an average wine contains up to 15% alcohol and is stored under 20 °C. Nevertheless, at higher temperatures, the stability of resveratrol is decreased [102].

The encapsulation of resveratrol within various matrices could increase its solubility and stability in wines. Several emulsion-based or liposome/niosome-based inclusion complexes and biopolymer-particles have been studied to overcome instability, hydrophobicity, and low bioavailability of resveratrol [100]. The use of encapsulated resveratrol can be used to produce “functional” wines, with enhanced resveratrol content, to ensure the benefits of their consumption for a long time after production and bottling. Nevertheless, encapsulated resveratrol probably cannot be used for the antioxidant and antimicrobial protection of wine, since in such form the bioactive compound is isolated from oxygen, light, yeasts or bacteria.

## 11. Potential Sources of Resveratrol for Commercial Production

Consumer increase in the perception of functional foods [103], those that offer additional health benefits [104], made a significant impact on the research on plant extraction and chemical or biological synthesis of resveratrol. Chemical synthesis provides a good alternative towards the production of resveratrol; the yields are relatively high, though the synthesis protocols are rather complex with many unwanted byproducts that limit the application of resveratrol derived in this way [105,106].

**Figure 1:**
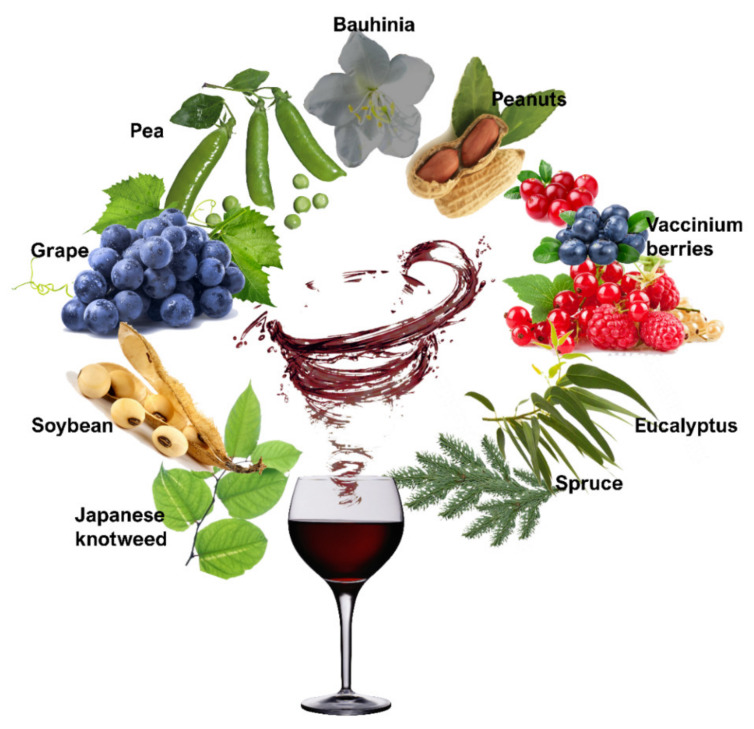
Visual representation of resveratrol sources to create enriched types of wine.

Extraction from plants (Figure 1) seems to be an excellent alternative to chemical synthesis. Resveratrol has been detected in more than 70 plant species [107] from 9 plant families [108]. Major sources include peanuts (*Arachis hypogaea*), grapes (*V. vinifera*) and grape products (must, wine), soybean (*Glycine max*) [108], pea (*Pisum sativum*) [109], berries (*Vaccinium* spp.) [110], Japanese Knotweed (*Fallopia japonica*) [111], spruce (*Picea excelsa*) [112], bauhinia (*Bauhinia racemosa*), and eucalyptus (*Eucalyptus sp.*) [113]. In the grapevine, resveratrol occurs mainly in grape skin, as well as in seeds, leaves, petioles, cluster stems, and roots of the plant [52,53,114]. Another important source for the isolation of resveratrol seems to be the canes of the vine; large quantities of these are removed each year during pruning. The utilization of canes for resveratrol extraction appears to be quite an economical source, as it have been found to contain up to 5mg of trans-resveratrol per gram of dry weight [115]. Following the vast source diversity, today, more than 92 resveratrol compounds have been identified [107], including monomers, as well as dimers, trimers, tetramers, hexamers, pentamers, and octamers of resveratrol [108].

The extraction of these molecules from plant sources is one of the strategies to cover the increasing market demand. The isolation or purification of natural products produces low concentration levels of the phytochemicals, as opposed to high plant biomass, which also contains unwanted side-products [116]. Moreover, these processes are time-consuming, expensive, wasteful regarding natural resources, and environmentally unsafe due to the excessive usage of solvents. The industry needs alternatives to plant extraction approaches while reducing costs at the same time. On the other hand, chemical synthesis offers an alternative procedure, but the chemical complexity of natural products makes it unfeasible to synthesize purified bioactive molecules combining low-cost production [117]. Thus, alternative approaches were sought following the development of synthetic biology to overcome the above-mentioned chemical synthesis and plant isolation drawbacks [118].

## 12. Innovative Microbial Biosynthesis of Resveratrol through Metabolic Engineering

Resveratrol, a stilbene, is a non-flavonoid polyphenolic compound coming from the branch of secondary metabolism responsible for the biosynthesis of phenylpropanoids [10,11]. Several works report the use of plant tissues or cells to produce resveratrol, but these systems, although managing to produce high yields [119,120], comprise insurmountable disadvantages, e.g., the complexity of the used media or cell growth speed that may affect the economics of the production.

In most of the microbial systems, the primary energy source is glucose that enters in the cell and is sequentially transformed towards the biosynthesis of aromatic amino acids, the starting molecules of phenylpropanoid metabolites. The significance of stilbenes on plant and human welfare [10] resulted in the proposal of a diverse array of strategies employing recombinant DNA technology to bio-transform simple and cheap precursor molecules to the more complex core molecule of resveratrol and its derivatives [117]. Since the first attempt at overexpressing pivotal genes of the phenylpropanoid metabolism in *Escherichia coli* [121], or the first attempt at producing resveratrol heterologously [106], a plethora of works has been reported either to produce resveratrol or natural and unnatural forms of it [10,11].

**Figure 2:**
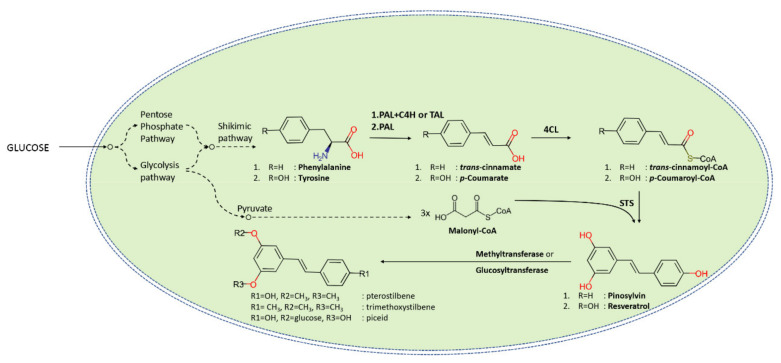
Representation of the biosynthetic pathway that transforms phenylalanine or tyrosine coming from primary metabolism into selected stilbenoids.

In nature, stilbenes are produced by a branch of phenylpropanoid metabolism, which typically leads to the production of flavonoids, anthocyanins, or lignins [10,117]. Either phenylalanine or tyrosine is deaminated by specific ammonia lyases (phenylalanine or tyrosine ammonia lyase, PAL, or TAL, respectively) to produce p-coumaric acid which is further activated by the ligation with coenzyme A (CoA) to generate the p-coumaroyl-CoA (Figure 2). At that point of the main phenylpropanoid pathway, resveratrol synthase acts on the activated p-coumaric acid utilizing three molecules of malonyl-CoA as co-factors to produce resveratrol [10], the first and most famed stilbene compound.

The above-mentioned resveratrol biosynthetic pathway fed directly from products of the primary metabolism encompassing PAL or TAL have been heterologously expressed into microorganisms to produce up to 531.41 mg/L of resveratrol or 70 mg/L of pinosylvin (Table 4). Attempts to produce resveratrol by supplementation of intermediate compounds have been published [122,123,124,125,126,127], but is out of the scope of this review. The first attempt to produce resveratrol in *S. cerevisiae* from phenylalanine was that of Trantas et al. [9], followed by efforts to achieve better efficiencies in yeast or bacteria [128]. Since then, following different approaches and optimization efforts has increased production levels. Shin et al. increased the resveratrol production level by the increase of internal malonyl-CoA pool [129]. Wang et al. increased the level of productivity when they utilized a codon-optimized TAL and a fused DNA fragment coding for both 4CL and RS gene activities (4CL:RS) [130]. The TAL gene accepted tyrosine instead of phenylalanine as a substrate, and thus there was no need for the activity of a CPR-dependent C4H. With such a strategy, the utilization of codon-optimized genes and fused proteins, the four needed biochemical activities were performed by only two expressed DNA sequences.

An interesting approach was followed by Camacho-Zaragoza et al. [132], who applied a co-cultivation strategy of two *E. coli* engineered strains; the first was able to transform the glycerol-supplemented medium into p-coumaric acid while the second was able to transform the p-coumaric acid into resveratrol at a fairly high concentration of 22.6 mg/L. However, a great step forward was made by Li et al. [133] when they achieved chromosomal integration of three genes for the heterologous biosynthesis of resveratrol and three genes to enhance the native tyrosine production. That way, utilizing a fed-batch approach in a bioreactor, they reached a final titer of 41,565 mg/L directly from glucose (53,141 mg/L from ethanol).

Naturally, there are many stilbene derivatives produced from reactions that decorate the basic stilbene backbone (e.g., resveratrol or pinosylvin) producing methylated, glycosylated or prenylated forms of them. Those compounds have been found to possess properties competitive to the popular resveratrol [141].

Recently, efforts have been focused on the modification of resveratrol to increase potency for active molecules. For this purpose, genes serving as decorative agents of secondary metabolites were cloned from various sources. These enzymes have evolved to possess less stringent substrate specificity and thus are good candidates for resveratrol-modifying enzymes. Kang et al. tested various O-methyltransferases from *Sorghum bicolor* on their ability to perform a varying degree of methylation on the hydroxyl groups of resveratrol [137] to produce pterostilbene or trimethoxystilbene. On another work, Choi et al. constructed an artificial biosynthetic pathway to produce a mixture of three resveratrol glucosides [138]. Utilizing an enzyme that had been earlier characterized as a UDP-glucosyltransferase able to add glucose sugars to various flavonoids, they managed to synthesize up to 11.7 mg/L of *trans*-piceid, *cis*-piceid, and resveratrol 4′-*O*-glucoside in total.

Two alternative approaches to produce resveratrol were proposed by Wang et al. [142], Conrado et al. [143], and Katsuyama et al. [144]. The former two groups studied different synthetic scaffold approaches to maximize resveratrol production while the latter took advantage of a stilbene-producing *E. coli* strain to produce unnatural compounds by feeding with carboxylic acids other than p-coumaric acid. All the above-mentioned groups did not manage to reconstitute the full resveratrol pathway but are of extreme significance since they could lead to the optimization of the utilized systems.

Although the most used platforms for the heterologous production of resveratrol compounds are those utilizing the bacterium *E. coli* or the yeast *S. cerevisiae*, other platforms have also been reported. *Corynebacterium glutamicum*, a common species for the production of amino acids [145], was genetically modified to yield 12 mg/L of resveratrol [134,135]. A human cell line has also been tested; when the resveratrol pathway was introduced into HEK293 cells, de novo biosynthesis was detected, leading to intracellular accumulation of resveratrol reaching a concentration of 0.0283 mg/L [131]. Both attempts did not achieve high yields, but they showed that using a fusion protein in mammalian cells may provide additional opportunities for medical and pharmaceutical research. The bacterium *Streptomyces venezuelae* has also been utilized as an alternative host for resveratrol production but not with the full heterologous pathway [146,147].

## 13. Alternative Biotechnological Production of Resveratrol through Plant Cell Suspensions

The cultivation of plant cells in a way similar to the cultivation of microbes is an alternative way to produce secondary metabolites and more specifically resveratrol [148]. Donnez et al. refer to the ability of *V. vinifera* suspension cultures (cv. Monastrell) to produce up to 5000 mg/L of resveratrol, when specific b-cyclodextrins elicitors were utilized in laboratory-scale small Erlenmeyer flasks [149]. Similar attempts were made by Belchi-Navarro et al. who managed to produce 2140 mg/L of resveratrol but also studied the effect of several parameters on resveratrol production [139]. More recently, Lambert et al. produced 4230 mg/L of resveratrol utilizing *V. vinifera* cv. Labrusca cells elicited by methyl jasmonate and methyl-β-cyclodextrins in a 20 L bioreactor or 6141 mg/L in Erlenmeyer flasks [140].

## 14. Conclusions

Resveratrol appears to be of great interest in the effort to replace or reduce the addition of sulfites in wine. In contrast to sulfites, it has a positive impact on consumer health. However, its content in a typical wine varies as a result of the difference in extraction efficiency from the skins and seeds during crashing and must preparation. The appropriate concentration of resveratrol in wine can be achieved by adding in the must the required amount before or after fermentation, leading to the production of resveratrol-enriched wines. However, extraction from plants has serious drawbacks, thus limiting its exploitation in wine industry. To meet the above needs, efforts are being made to produce resveratrol through alternative innovative ways.

Various synthetic and molecular biology tools facilitated scientists to construct microbial factories able to transform cheap precursor molecules to resveratrol or resveratrol-derived molecules to a level around 0.5 g/L. Although the efficiency is not impressive, optimization efforts or the use of bioreactors will substantially increase production levels. On the other hand, a well-designed *V. vinifera* cell suspension approach may provide an alternative system for the over-production of resveratrol that can surpass the threshold of 6 g/L. Since science has already entered the OMICS era (referring to the comprehensive analysis of complete genetic or molecular profiles of organisms), the parallel processing of genomics, transcriptomics, and metabolomics data will identify new routes towards the increase of production levels.

## Figures and Tables

**Table 1:** Upper limits for total sulfur dioxide (SO_2_) in wines in major winemaking regions worldwide.

Region	Limit	Regulatory
Australia	250 mg/L for wines containing <35 g/L sugars 300 mg/L for wines containing ≥35 g/L sugars	Australia New Zealand Food Standards Code–Standard 4.5.1: Clause 5(5)(a)
Canada	70 mg/L for all wines (free SO_2_) 350 mg/L for all wines (total SO_2_)	Can. Food and Drug Regulations (C.R.C., c. 870), B.02.100
European Union	150 mg/L for red wines (containing ≤5 g/L of sugar content) 200 mg/L for white and rosé wines (containing ≤5 g/L of sugar content) 200 mg/L for red wines (containing >5 g/L of sugar content) 250 mg/L for white and rosé wines (containing >5 g/L of sugar content) 300 mg/L exceptionally in certain wines, described in Reg. (EU) 2019/934	Regulation (EU) 2019/934
India	450 mg/L for all wines	The Prevention of Food Adulteration Act & Rules
Japan	350 mg/Kg for all wines	Japan′s Specifications and Standards for Food Additives
New Zealand	250 mg/Kg for wines containing <35 g/L sugars 400 mg/Kg for wines containing ≥35 g/L sugars	Australia New Zealand Food Standards Code–Standard 4.5.1: Clause 5(5)(a)
South Africa	160 mg/L for white wines (containing <5 g/L of sugars) 150 mg/L for red wines (containing <5 g/L of sugars) 200 mg/L for all wines (containing ≥5 g/L of sugars) 300 mg/L exceptionally for noble late harvest wine and wine from naturally dried grapes	Liquor Products Act 60 Regulation 32, Table 8, Note 2
United States of America	350 mg/L for all wines (total SO_2_)	Code of Federal Regulations, 27 CFR § 4.22
World–International Organisation of Vine and Wine (OIV)	150 mg/L for red wines (containing ≤4 g/L of reducing substances) 200 mg/L for white and rosé wines (containing ≤4 g/L of reducing substances) 300 mg/L for red, rosé and white wines (containing >4 g/L of reducing substances) 400 mg/L exceptionally in certain sweet white wines	International Code of Oenological Practices (Issue 2019)

**Table 2:** *trans*-Resveratrol content of white, rosé, and red wines, from the major winemaking countries worldwide.

Country	Wine Color	*trans*-Resveratrol (mg/L)		Sample Number	Reference
		Range	Mean		
Australia	Red	0.100–0.950	0.440	5	[54]
	Red	1.461–1.548	1.504	2	[55]
	Red		2.371	35	[56]
Brazil	Red	0.820–5.750	2.570	36	[57]
California	Red	0.226–2.319	0.864	8	[55]
	Red		1.685	72	[56]
France	Red	1.735–2.901	1.966	4	[55]
	Red		2.085	48	[56]
Greece	Red	0.550–2.534	1.105	29	[58]
	Red	0.325–1.569	0.873	15	[59]
	White	0.026–0.142	0.043	15	[59]
	Red	0.352–1.991	0.895	13	[60]
	White	0.005–0.571	0.229	18	[60]
Hungary	Red	0.100–14.300	2.380	68	[61]
	White	0.200–0.780	0.563	3	[61]
Italy	Red	0.657–1.155	0.984	3	[55]
Spain	Red	0.320–4.440	1.471	74	[62]
	Rosé	0.120–2.800	0.669	24	[62]
	Red	0.600–8.000	2.485	18	[63]
	Red	<0.012–0.472	0.179	14	[64]
	White	<0.012–0.062	0.024	8	[64]
Turkey	Red	0.176–4.403	1.203	7	[65]
	White	0.116–1.243	0.891	4	[65]

**Table 3:** Minimum inhibitory concentration (MIC) of resveratrol [mg/L] on wine-related microorganisms.

	Species	MIC (mg/L)	Reference
Yeasts	*Debaryomyces hansenii*	250	[96]
	*Dekkera bruxellensis*	256	[95]
	*Hanseniaspora uvarum*	256	[95]
	*Saccharomyces bayanus*	250	[96]
	*S. cerevisiae*	250	[96]
		256	[95]
	*S. cerevisiae × S. kudriavzevii × S. bayanus*	250	[96]
	*Wickerhamomyces anomalus*	250–500	[96]
	*Zygosaccharomyces bailii*	256	[95]
	*Z. rouxii*	512	[95]
Acetic Acid Bacteria	*Acetobacter acetii*	250	[96]
		256	[95]
	*A. oeni*	256	[95]
	*A. pasteurianus*	256	[95]
	*Gluconobacter cerinus*	>1000	[96]
Lactic Acid Bacteria	*Lactobacillus hilgardii*	250 855	[96] [94]
	*L. plantarum*	250	[96]
	*Oenococcus oeni*	250	[96]
		307–698	[94]
	*Pediococcus parvulus*	250	[96]
	*P. pentosaceus*	715	[94]

**Table 4:** Recorded reports of resveratrol production utilizing precursor molecules derived from primary metabolism (e.g., phenylalanine or tyrosine) or added. NA, not added; GO, gene overexpression; F, utilization of fussed enzymes; OPT, utilization of codon-optimized genes; TRA, utilization of a transporter, GOCI, gene expression from chromosomal integration; BIOR, fed-batch fermentation in controlled bioreactor; OPM, overexpression of genes of the primary metabolism; IPCO, increase the pool of implicated co-factors; MOC, utilization of a monoculture approach; COC, utilization of a co-culture approach; KOG, knock-out of specific genes of the primary metabolism; CER, addition of cerulenin; NE, not estimated; NA, not applied.

End Product	Precursor Molecule (Alternative Source)	Number of Genes	Target Genes and Sources	Strategy	Host Organism	Production Level (mg/L)	Reference
Resveratrol	NA	3	*Rhodobacter sphaeroides* (TAL), *Arabidopsis thaliana* (4CL), *Vitis vinifera* (RS)	MOC GO FUS	Human HEK293 cells	0.0283	[131]
Resveratrol	Phenylalanine	5	*Populus trichocarpa* x *P.deltoides* (PAL, CPR), *Glycine max* (C4H, 4CL), *Vitis vinifera* (RS)	MOC GO	*Saccharomyces cerevisiae*	0.29	[9]
Resveratrol	Galactose	3	*Rhodobacter sphaeroides* (TAL), *Arabidopsis thaliana* (4CL), *Vitis vinifera* (RS)	MOC GO OPT FUS	*S. cerevisiae*	1.06	[130]
Resveratrol	Tyrosine	3	*Saccharothrix espanaensis* (TAL), *Streptomyces coelicolor* (4CL), *Arachis hypogaea* (RS)	MOC GO OPT	*Escherichia coli*	1.4	[128]
Resveratrol	Galactose (Tyrosine)	4	*Rhodosporidium toruloides* (PAL), *A. thaliana* (C4H), *A. thaliana* (4CL), *A. hypogaea* (RS), *S. cerevisiae* (ACC1)	MOC GO IPCO	*S. cerevisiae*	4.3 (5.8)	[129]
Resveratrol	Glycerol	5	*E. coli* (AroG, tktA)*, Rhodothorula glutinis* (TAL), *S. coelicolor* (4CL), (STS), *ΔpheA*	COC GO OPM KOG	*E. coli*	22.6	[132]
Resveratrol	Glucose (Ethanol)	6	*Herpetosiphon aurantiacus* (TAL)*, Arabidopsis thaliana* (4CL), *Vitis vinifera* (RS)*, S. cerevisiae* (ARO4)*, S. cerevisiae* (ARO7), *S. cerevisiae* (ACC1)	MOC GOCI BIOR OPM IPCO	*S. cerevisiae*	415.65 (531.41)	[133]
Resveratrol	Glucose	4	*E. coli* (AroH) *Flavobacterium johnsoniae* (TAL) *A. hypogaea* (STS), *Petroselinum crispum* (4CL), *ΔqsuB*	MOC KOG OPT GOCI GO	*Corynebacterium glutamicum*	12	[134,135]
Pinosylvin	Glucose (phenylalanine)	3	*P. crispum (PAL)*, *S. coelicolor* (4CL), *Pinus strobus (STS)*	MOC OPT GO CER	*E. coli*	70 (91)	[136]
Methylated Resveratrol derivatives	Glucose	5	*S. espanaensis* (TAL), *Streptomyces coelicolor* (4CL), *Stilbene synthase* (STS), *Sorghum bicolor* (OMT),	MOC GO OPT	*E. coli*	NE	[137]
Mix of 3 glucosylated Resveratrol derivatives	Glucose	4	*S. espanaensis* (TAL), *S. coelicolor (4CL)*, *Arachis hypogaea (STS)*, *Bacillus licheniformis* (YjiC)	MOC GO OPT	*E. coli*	11.7	[138]
Resveratrol	NA	NA	NA	Cell suspension	*V. vinifera* cv. Monastrell	2140	[139]
Resveratrol	NA	NA	NA	Cell suspension in flasks (Bioreactor)	*V. vinifera* cv. Labrusca	6141 (4230)	[140]
